# Metagenomic analysis reveals a functional signature for biomass degradation by cecal microbiota in the leaf-eating flying squirrel (*Petaurista alborufus lena*)

**DOI:** 10.1186/1471-2164-13-466

**Published:** 2012-09-10

**Authors:** Hsiao-Pei Lu, Yu-bin Wang, Shiao-Wei Huang, Chung-Yen Lin, Martin Wu, Chih-hao Hsieh, Hon-Tsen Yu

**Affiliations:** 1 Institute of Zoology and Department of Life Science, National Taiwan University, Taipei, Taiwan, ROC; 2 Institute of Information Science, Academia Sinica, Taipei, Taiwan; 3 Department of Biology, University of Virginia, Charlottesville, VA, 22904, USA; 4 Institute of Oceanography, National Taiwan University, Taipei, Taiwan; 5 Institute of Ecology and Evolutionary Biology, National Taiwan University, Taipei, Taiwan; 6 Genome and Systems Biology Degree Program, National Taiwan University, Taipei, Taiwan, ROC

**Keywords:** Coevolution, Gut microbiota, Folivore, Metagenomics, Fosmid

## Abstract

**Background:**

Animals co-evolve with their gut microbiota; the latter can perform complex metabolic reactions that cannot be done independently by the host. Although the importance of gut microbiota has been well demonstrated, there is a paucity of research regarding its role in foliage-foraging mammals with a specialized digestive system.

**Results:**

In this study, a 16S rRNA gene survey and metagenomic sequencing were used to characterize genetic diversity and functional capability of cecal microbiota of the folivorous flying squirrel (*Petaurista alborufus lena*). Phylogenetic compositions of the cecal microbiota derived from 3 flying squirrels were dominated by *Firmicutes*. Based on end-sequences of fosmid clones from 1 flying squirrel, we inferred that microbial metabolism greatly contributed to intestinal functions, including degradation of carbohydrates, metabolism of proteins, and synthesis of vitamins. Moreover, 33 polysaccharide-degrading enzymes and 2 large genomic fragments containing a series of carbohydrate-associated genes were identified.

**Conclusions:**

Cecal microbiota of the leaf-eating flying squirrel have great metabolic potential for converting diverse plant materials into absorbable nutrients. The present study should serve as the basis for future investigations, using metagenomic approaches to elucidate the intricate mechanisms and interactions between host and gut microbiota of the flying squirrel digestive system, as well as other mammals with similar adaptations.

## Background

Although ancestors of mammals are believed to have been small carnivores, primarily feeding on invertebrates or other vertebrates [1], dietary shifts into herbivorous niches may have been critical for the massive expansion of mammals [2]. The symbiotic relationship of gut microbiota to provide metabolic activities lacking in the host was undoubtedly a great success in mammalian evolution [3]. Each animal operates as a “super-organism”, which consists of gene functions from its own genome, as well as those of the gut microbiome [4]. Although the latter enable the host to exploit new dietary niches, the paucity of well characterized model systems has limited understanding of the diversity of gut microbial ecosystems and interactions among components of the “super-organism.” In particular, a complex gut microbiota would be expected in highly folivorous animals, since this specific foraging habit was presumably facilitated by adaptive evolution to extract energy from fibrous leaves.

The diet of giant flying squirrels (genus *Petaurista*), which are adapted to a leaf-eating niche in forest trees in the montane areas of Taiwan, primarily consists of leaf parts (buds, petioles, young leaves, and mature leaves) of diverse tree species [5,6]. That those leaves supply less energy per unit weight relative to other plant parts (e.g. fruits, flowers and seeds) poses special difficulties for folivores [7]. Furthermore, the giant flying squirrel weighs no more than 1.5 kg, making it one of the smallest mammals sustained by a strictly folivorous diet [8]. Compared to large herbivores (e.g. ruminants, horses and elephants), small herbivores have a relatively high energy demand, but low absolute gut capacity [9]. Therefore, giant flying squirrels are expected to have complex digestive strategies, including a well-adapted gut microbiota.

Metagenomics, which uses efficient sequencing techniques to provide enormous datasets for phylogenetic and functional analyses, is well suited to investigating gut microbiota engaged in complex metabolic interactions [10,11]. In the present study, a fosmid library was used for reconstructing partial genomes of novel uncultured bacteria expected to be involved in plant biomass degradation. Furthermore, a 16S rRNA gene survey and metagenomic approaches were used to investigate genetic diversity and functional capability of the cecal microbiota in the folivorous flying squirrel (*Petaurista alborufus lena*).

Our data clearly elucidated the functional signature of this mammalian "super-organism" adapted to a particular ecological environment. During the transition to a specific foliage diet, extensive changes due to adaptive evolution on flying squirrels and their gut microbiota were manifested in the entire system, rather than a single species or gene. We inferred that the limited energy provided by a leaf diet was allocated and circulated among numerous microbial species and the host, apparently resulting in mutually beneficial interactions. The metagenomic datasets generated advanced our understanding regarding the complex processes of supplying the energy needed for small mammalian folivores; furthermore, they may provide insights into energy transfer in forest ecosystems.

## Results

### Anatomical confirmation of the cecum as a fermentation chamber

It is generally accepted that small mammalian herbivores have substantial cecal microbial fermentation [7]. We sought to verify if this was the case in the flying squirrel. We examined 4 white-faced flying squirrels, each with a full gastrointestinal (GI) tract. For all 4 squirrels, the average length of the entire GI tract was 411 ± 35 cm (mean ± SD), 10 times the body length (average, 40 ± 3 cm). This GI tract to body length ratio was similar to those of other cecum-fermenter mammals [9], such as rabbits (ratio of 10) and lemurs (ratio of 13) [12]. The weight/length ratio including food (g/cm) was used as an indicator of the digesta-retaining capacity of the small intestine, cecum, and large intestine. An extremely distended cecum, containing nearly 50% of the gut contents by weight, was the most salient feature (Table 1). Moreover, the weight/length ratio for the cecum was 6–8 times greater than that of the small or large intestines.

**Table 1:** Mean ± SD anatomical features of 3 intestinal compartments of the white-faced flying squirrel (N = 4)

	**Small intestine**	**Cecum**	**Large intestine**
Weight (g)	81.75 ± 18.93	143.00 ± 31.51	66.00 ± 12.41
Length (cm)	182.75 ± 28.44	48.53 ± 2.07	171.95 ± 7.76
W/L (g/cm)	0.45 ± 0.08	2.93 ± 0.56	0.38 ± 0.06

### Phylogenetic profiles of cecal microbiota, based on 16S rRNA gene sequences

To characterize the bacterial community of the cecum, 16S rRNA gene libraries were constructed from 2 individuals (FS1 and FS2). After elimination of short, low-quality, and chimera sequences, a total of 520 and 440 sequences were obtained for FS1 and FS2, respectively. Based on a 97% sequence identity threshold, the 2 libraries respectively contained 173 (FS1) and 165 (FS2) phylotypes or OTUs (Operational Taxonomic Units), with 262 (FS1) and 293 (FS2) estimated species diversity (Chao1) of cecal microbiota ( Additional file 1).

The 16S rRNA sequences from the 2 flying squirrels were classified into 4 phyla of bacteria, with <1% unclassified bacterial sequences (Table 2). Two microbial communities were both extremely dominated by *Firmicutes*, with sequence abundances of 96.5 and 88.4%, respectively (average, 92.92%). The remainder of the sequences belonged to *Actinobacteria* (2.7 and 5.9%; average, 4.17%), *Proteobacteria* (0.6 and 1.6%; average, 1.04%), and *Verrucomicrobia* (0 and 3.2%; average, 1.46%).

**Table 2:** Comparison of the phylogenetic composition of bacteria

**Bacterial phylum**	**Flying squirrel**					**Mouse**				**Cattle**			
	**OTU**	**OTU (%)**	**Clones**	**Clones (%)**		**OTU**	**OTU (%)**	**Clones**	**Clones (%)**	**OTU**	**OTU (%)**	**Clones**	**Clones (%)**
*Acidobacteria*	0	0.00	0	0.00	(0.48)	0	0.00	0	0.00	0	0.00	0	0.00
*Actinobacteria*	9	3.60	40	4.17	(8.19)	6	1.71	20	1.79	1	0.15	1	0.04
*Aquificae*	0	0.00	0	0.00	(0.06)	0	0.00	0	0.00	0	0.00	0	0.00
*Bacteroidetes*	0	0.00	0	0.00	(2.60)	56	15.95	325	29.02	92	13.63	347	12.34
*Chlamydiae*	0	0.00	0	0.00	(0.00)	0	0.00	0	0.00	1	0.15	1	0.04
*Chlorobi*	0	0.00	0	0.00	(0.45)	0	0.00	0	0.00	0	0.00	0	0.00
*Chloroflexi*	0	0.00	0	0.00	(1.77)	0	0.00	0	0.00	0	0.00	0	0.00
*Cyanobacteria*	0	0.00	0	0.00	(1.48)	0	0.00	0	0.00	0	0.00	0	0.00
*Deinococcus-Thermus*	0	0.00	0	0.00	(0.39)	0	0.00	0	0.00	0	0.00	0	0.00
*Firmicutes*	231	92.40	892	92.92	(60.78)	284	80.91	764	68.21	554	82.07	2083	74.08
*Fusobacteria*	0	0.00	0	0.00	(0.80)	0	0.00	0	0.00	0	0.00	0	0.00
*Planctomycetes*	0	0.00	0	0.00	(0.06)	0	0.00	0	0.00	2	0.30	2	0.07
*Proteobacteria*	5	2.00	10	1.04	(11.85)	3	0.85	9	0.80	7	1.04	352	12.52
*Spirochaetes*	0	0.00	0	0.00	(1.48)	0	0.00	0	0.00	0	0.00	0	0.00
*Synergistetes*	0	0.00	0	0.00	(0.00)	0	0.00	0	0.00	1	0.15	1	0.04
*Thermotogae*	0	0.00	0	0.00	(0.77)	0	0.00	0	0.00	0	0.00	0	0.00
*Verrucomicrobia*	1	0.40	14	1.46	(8.77)	0	0.00	0	0.00	2	0.30	2	0.07
Candidate division OP10	0	0.00	0	0.00	(0.00)	0	0.00	0	0.00	1	0.15	1	0.04
Candidate division TM7	0	0.00	0	0.00	(0.00)	1	0.28	1	0.09	6	0.89	9	0.32
Unclassified bacteria	4	1.60	4	0.42	(0.06)	1	0.28	1	0.09	8	1.19	13	0.46
Total	250	100.00	960	100.00	(100.00)	351	100.00	1120	100.00	675	100.00	2812	100.00

“Flying squirrel” represented combined data for cecal mircrobiota of 2 flying squirrels (FS1 and FS2). "Mouse" represents combined data for cecal microbiota of 3 lean mice [14]. "Cattle" represents combined data for rumen microbiota of 3 cattle [15]. Numbers of phylotypes (OTUs) for each phylum were given for analyses of 16S rRNA gene libraries. Numbers in parentheses for “Flying squirrel” were relative abundances estimated by fosmid end-sequences from 1 flying squirrel (FS5).

**Figure 1:**
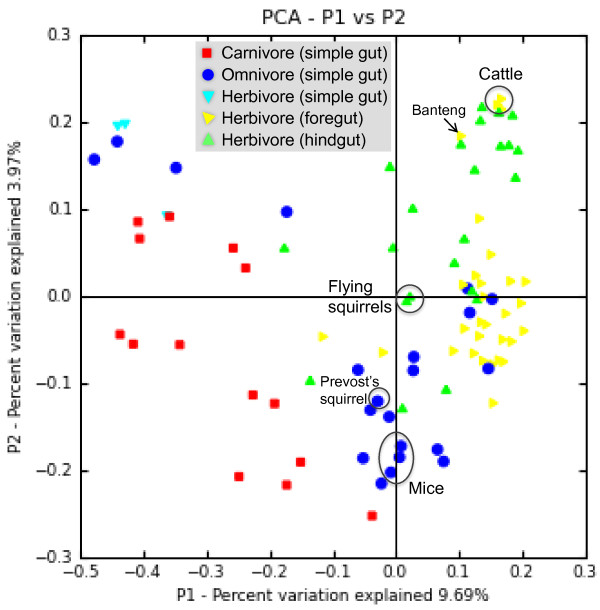
**Relationships of gut bacterial communities using principal coordinates analysis (PCoA) of the UniFrac metric matrix.** Data included sequences from fermentation chambers (flying squirrels, cattle and mice) and from mammalian fecal samples [13]. The scores for the first 2 dimensions (P1 and P2) are plotted. Data for the cattle and mice were derived from [15] and [14], respectively.

Data from the present study were compared to published data from fecal samples of 56 mammalian species [13], and from the fermentation chambers of lean laboratory mice (cecum) [14] and cattle (rumen) [15], using the principal coordinates analysis (PCoA) of the UniFrac metric matrix (Figure 1). This analysis summarized variation in sampled communities, based on phylogenetic differences in bacterial members, and generated plots that separated individual communities. The flying squirrels were near to other herbivores, but not clustered with the omnivorous Prevost's squirrel, although they are phylogenetic kin (Figure 1). As expected, mice were similar to other omnivores, whereas cattle were far from most foregut herbivores, as were banteng, a close relative of cattle, which may reflect domestication of these two ruminant species.

To gain more insight into fermentation chambers (functional counterparts to the flying squirrel’s cecum), we further compared our data to those from the mouse cecum [14] and the cattle rumen [15] (Table 2 and Additional file 2). A total of 11 bacterial phyla/groups were identified by 16S rRNA gene sequences obtained from the 3 host species (Table 2), of which microbial communities differed in the proportions of microbial groups (*P* < 0.001, *Χ*^2^). It was noteworthy that 3 communities were all dominated by *Firmicutes* (flying squirrel 93%, mouse 68%, and cattle 74%). Further, *Bacteroidetes* was absent from the flying squirrel, but was well represented in both the mouse (29%) and cattle (12%). When the 16S rDNA sequence variation and relative abundances of phylotypes were considered, the 3 species, which each formed a tight cluster, were well separated by PCoA (first 2 axes summarized 71.7% of total variation), based on the weighted UniFrac metric matrix ( Additional file 2).

### Phylogenetic profile of microbiota based on fosmid end-sequences

Based on analysis of ~3 Mb of metagenomic sequences (from FS5), 5,012 open reading frames (ORFs) were predicted from the fosmid end-sequences and treated as gene tags (for further annotation). Up to 65% of the gene tags were classified into taxonomic ranks, based on matches in the SEED database. According to the annotation, the majority of the microbiota belonged to Bacteria (95.8%), with the remainder attributed to Archaea (3.6%), Eukaryota (0.5%), and Viruses (0.1%).

The annotation allowed an additional assessment of microbial diversity from a third individual (FS5) in the present study. For bacteria, the most abundant phylum was *Firmicutes* (61%), followed by *Proteobacteria* (12%), *Verrucomicobia* (9%), *Actinobacteria* (8%),*Bacteroidetes* (3%), *Chloroflexi* (2%), *Spirochaetes* (1%), *Cyanobacteria* (1%), with an additional 8 phyla/groups each constituting < 1% (Table 2). In general, predominant phylogenetic groups represented by the fosmid end-sequences were similar to those identified in the 16S rRNA gene survey, but the pattern, based on fosmid end-sequences, differed from that based on 16S rRNA sequences (*P* < 0.001, *Χ*^2^), as 16S probing could only detect bacterial phyla and more bacterial phyla were detected by fosmid end-sequences (Table 2), including those that were likely missed due to primer bias resulting from the 16S rRNA gene survey. Additionally, fosmid end-sequences detected non-bacterial phyla and viruses.

One hundred and sixteen sequences were assigned to archaea, namely *Euryarchaeota* (92%) and *Crenarchaeota* (8%); the majority belonged to methanogens (e.g. *Methanomicrobia*, *Methanobacteria*, *Methanococci*, and *Methanopyri*). Sixteen eukaryotic sequences were also identified in the cecal microbiome, belonging to multicellular metazoan (possibly host DNA debris), Fungi, and Viridiplantae (likely dietary debris). Finally, 3 viral sequences were identified; all were assigned to double-stranded DNA viruses (a phage family: *Siphoviridae*) which only infect bacteria.

### Functional profile of the microbiota, based on fosmid end-sequences

**Figure 2:**
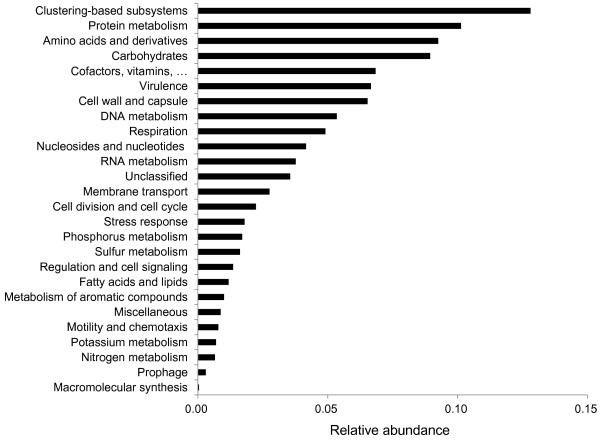
Functional profile of the cecal microbiota of the flying squirrel according to the SEED Subsystem Hierarchy 1.

The gene functions of the cecal microbiota were analyzed by searching similarity against several databases. Based on the MG-RAST results, 2,280 of the 5,012 gene tags were assigned to 1 of the SEED subsystems, in which genes are annotated according to biochemical pathways and their specific functional roles [16]. On the basis of SEED Subsystem Hierarchy 1, hits were attributed to 26 functional groups (Figure 2). The “clustering-based subsystems” was the largest group, representing ~13% of hits. Genes in this category are functionally coupled, since they usually cluster together in genomic regions, although their activities are poorly understood. The next 4 most prominent groups were involved in protein metabolism (10%), amino acids and derivatives (9%), carbohydrate metabolism (9%), and synthesis of cofactors / vitamins (7%). Collectively, these 5 dominant groups accounted for almost 50% of the hits.

Protein metabolism was the second most prominent functional category and was dominated by the subcategory of biosynthesis (69%), followed by folding (16%), secretion (8%), and degradation (6%). Within the protein biosynthesis subcategory, most genes were involved in tRNA aminoacylation (adding an amino acid to tRNA). In addition, bacterial ribosomal proteins (both small and large subunits) were also abundant in this subcategory. In the protein folding subcategory, 36 chaperone proteins (e.g. GroEL, GroES, and DnaJ) were identified. Proteins involved in the secretory pathway, e.g. preprotein translocase subunits (SecG and SecY) and protein-export membrane proteins (SecD and SecF), were also detected.

The third most prominent functional category contained genes involved in production and recycling of amino acids. In addition to those involved in a variety of biosynthetic pathways, genes related to urea hydrolysis, including genes coding for the alpha, beta, and gamma subunits of urease, and for urease accessory protein UreD / UreG, were also detected.

The fourth most prominent category, carbohydrate metabolism, was dominated by central carbohydrate metabolism (35%), including enzymes involved in the TCA cycle, pyruvate metabolism, and 3 pathways for glucose degradation to pyruvate (namely the Embden-Meyerhof, Entner-Doudoroff, and pentose phosphate pathways). In addition, the subcategories of monosaccharides (23%) and di- and oligosaccharides (14%) were also abundant. Both sugar-degrading enzymes (e.g. beta-glucosidase, beta-galactosidase, beta-xylosidase, and endoglucanase) and sugar-transporters (for xylose ribose, fucose, allose, rhamnose, arabinose, lactose, and cellobiose) were detected.

Following the carbohydrate metabolism category was a group of genes involved in synthesis of cofactors / vitamins, of which folate biosynthesis (24%) was the most abundant subsystem. In addition, syntheses of tetrapyrroles, coenzyme A, and quinone cofactors were well-represented (19, 13, and 12% of the category, respectively). Genes associated with biosynthesis of B vitamins, such as thiamine (B1), riboflavin (B2), niacin (B3), pantothenic acid (B5), pyridoxine (B6), biotin (B7), folic acid (B9), and cobalamin (B12), were also detected.

Similar to results obtained from the SEED subsystems, functional categories identified using the COG (Clusters of Orthologous Groups of proteins; Additional file 3) and KEGG (Kyoto Encyclopedia of Genes and Genomes; Additional file 4) databases showed that genes involved in amino acid metabolism (7 and 13%), carbohydrate metabolism (4 and 13%), and metabolism of cofactors and vitamins (4 and 4%) were common within the cecal metagenome. Comparing the proportion of major metabolic categories based on the SEED and KEGG databases, carbohydrate metabolism was as dominant as amino acid metabolism, whereas based on COG, amino acid metabolism was twice as well represented as carbohydrate metabolism. In addition, although SEED and COG showed that genes involved in metabolism of cofactors and vitamins were more abundant than those in nucleotide metabolism, KEGG showed the opposite trend. Some apparent discrepancies may be due to differences (among the 3 functional categorization schemes) in naming and assigning differences. According to the COG and KEGG classifications, genes involved in energy metabolism (7 and 6%) were abundant. Those genes were classified into SEED subsystems of respiration (5%), sulfur metabolism (2%), and nitrogen metabolism (1%). Otherwise, genes in protein metabolism of SEED were categorized into information processing groups such as translation of COG and KEGG databases.

To focus on carbohydrate-active enzymes related to degradation of polysaccharides, sequences were annotated using information from the CAZy database [17]. Thirty-three polysaccharide-degrading enzymes belonging to 16 glycoside hydrolase (GH) families and 1 carbohydrate esterase (CE) family were detected in the fosmid end-sequence dataset; 7 carbohydrate-binding modules (CBMs) associated with detected GHs were also identified (Table 3). These enzymes included cellulases (GH3 and GH9) and hemicellulases (GH2, GH35, GH39, and CE4). The amino acid identity between the fosmid end-sequences and the reference sequences ranged from 30 to 91%.

**Table 3:** Candidate fosmid clones containing enzymes for plant polysaccharide degradation

**Fosmid_ID**	**CAZy**	**Functional description**	**Identity (%)**	**CBM**
pLC07_F01	GH2	Beta-galactosidase	51	CBM32
pEB10_E10	GH3	Beta-glucosidase	38	
pEB09_F10	GH3	Beta-glucosidase	42	
pEB07_A10	GH3	Beta-N-acetylhexosaminidase	48	
pEB15_H04	GH9	Cellobiohydrolase	42	CBM3
pEB13_G01	GH13	4-alpha-glucanotransferase	36	
pLC07_G02	GH13	1,4-alpha-glucan branching enzyme	57	CBM48
pLC07_A12	GH18	Predicted glycosyl hydrolase	67	
pLC07_G03	GH18	Predicted glycosyl hydrolase	69	
pEA02_E01	GH20	Beta-N-acetylhexosaminidase	32	
pEB01_A03	GH20	Beta-N-acetylhexosaminidase	36	
pEB04_D05	GH20	Beta-N-acetylhexosaminidase	33	
pEA03_F02	GH23	Soluble lytic murein transglycosylase	82	
pEB14_G10	GH23	Soluble lytic murein transglycosylase	91	
pLC08_D10	GH23	Soluble lytic murein transglycosylase	88	
pLC09_B04	GH23	Soluble lytic murein transglycosylase	51	
pLD06_F11	GH23	Soluble lytic murein transglycosylase	91	
pLD10_C02	GH23	Soluble lytic murein transglycosylase	89	
pLD10_D11	GH29	Alpha-L-fucosidase	58	CBM32
pEB14_G12	GH29	Alpha-L-fucosidase	40	
pLC06_A10	GH29	Alpha-L-fucosidase	58	
pLC04_G11	GH31	Alpha-glucosidases	57	
pLD09_H03	GH33	Neuraminidase (sialidase)	37	
pLC03_A09	GH35	Beta-galactosidase	44	CBM32
pEB17_F07	GH39	Beta-xylosidase	38	
pLC08_F09	GH73	Glucosaminidase (YG repeat)	48	
pLD05_E02	GH73	Muramidase (flagellum-specific)	62	
pLC09_H11	GH77	4-alpha-glucanotransferase	33	
pLC09_B03	GH84	Hyaluronidase	46	CBM32
pLD05_D12	GH110	Alpha-1,3 galactosidase	49	CBM51
pEB16_A07	CE4	Predicted xylanase/chitin deacetylase	30	
pEB20_C02	CE4	Predicted xylanase/chitin deacetylase	31	
pLD04_D08	CE4	Predicted xylanase/chitin deacetylase	44	

### Gene contents of fosmid inserts containing carbohydrate-associated genes

**Figure 3:**
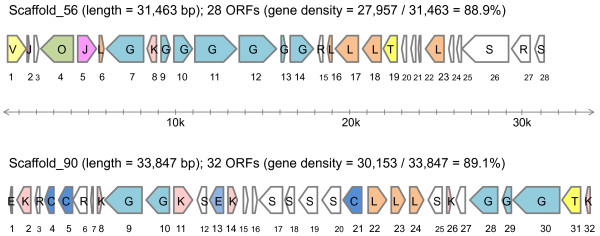
**Gene structures of 2 fosmid inserts: Scaffold_56 (GenBank: JQ335997) and Scaffold_90 (GenBank: JQ335998).** The ORFs are colored and labeled according to the COG functional categories as C (energy production and conversion), E (amino acid transport and metabolism), G (carbohydrate transport and metabolism), J (translation, ribosomal structure, and biogenesis), K (transcription), L (replication, recombination, and repair), O (posttranslational modification, protein turnover, chaperones), R (general function prediction only), S (function unknown), T (signal transduction mechanisms), and V (defense mechanisms). Further details of the putative function for each ORF are presented in Additional file 5.

Sequences from 100 fosmid inserts were characterized to provide a survey of large contiguous genomic fragments. A total of 157 Mb of pyrosequencing paired-end reads was assembled into 125 scaffolds, comprising 3,042 kb genomic fragments. The average scaffold length was 24 kb (range, 2 to 67). In this dataset, 2 large scaffolds (both > 30 kb), each containing at least 3 carbohydrate-active enzymes, were chosen for further analysis. The assembled sequences for these 2 fosmid inserts were 31,463 bp (Scaffold_56) and 33,847 bp (Scaffold_90) and contained 28 and 32 ORFs, respectively (Figure 3). On average, 89% of the sequences were protein-coding regions. The functional and taxonomic assignments of these ORFs were annotated according to the NCBI-nr and the COG databases ( Additional file 5).

Based on taxonomic assignments, these 2 genomic fragments were of bacterial origin and were likely derived from *Firmicutes* species, since approximately 90% of the ORFs were assigned to this phylum ( Additional file 5). Of the 60 ORFs in the 2 scaffolds, 33 had ≦ 60% identity with any known gene, whereas only 9 had ≧ 80% identity. We inferred that Scaffold_56 and Scaffold_90 represented segments of hitherto uncharacterized bacterial genomes. Based on the COG functional categories (Figure 3 & Additional file 5), 12, 8, and 7 ORFs were classified into the G (carbohydrate transport and metabolism), L (replication, recombination, and repair), and K (transcription) categories, respectively, with other categories containing ≦ 3 ORFs each.

As regards carbohydrate-active enzymes, 6 putative GHs were encoded by ORFs-7, 11, and 12 of Scaffold_56 and ORFs-9 and 28–30 of Scaffold_90 (Figure 3 and Additional file 5). With the exception of ORF-12 in Scaffold_56, which coded for a GH2 enzyme, all of these ORFs coded for members of the GH3 family. The identified GH2 contained a catalytic domain (PF02836) and a sugar-binding domain (PF02837) with potential activities as a beta-galactosidase, beta-mannosidase, or beta-glucuronidase. The ORF-28 and ORF-29 in Scaffold_90 coded for a polypeptide homologous to the C-terminal domain (PF01915) or N-terminal domain (PF00933) of a GH3 enzyme, respectively, whereas ORF-7 and ORF-11 in Scaffold_56 and ORF-9 and ORF-30 in Scaffold_90 each coded for both the N-terminal and C-terminal domains of GH3 enzymes with known activities, e.g. beta-glucosidase and beta-xylosidase.

**Figure 4:**
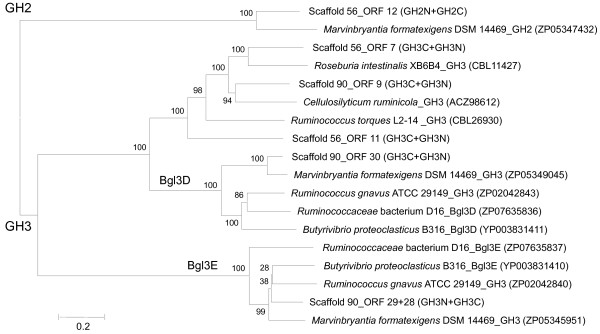
**Distance dendrogram of glycoside hydrolases.** Data included the deduced amino acid sequences of 6 GHs in Scaffold_56 and Scaffold_90 and their homologs from databases. The tree was constructed by the neighbor-joining method with 1,000 bootstrap replications using MEGA 5 software. Numbers near nodes indicate bootstrap values.

The protein sequences of the GHs and their homologs from databases were used to construct a gene dendrogram (Figure 4). The GH2 sequences were located at the root and were separated from the GH3 sequences. Three GH3 ORFs (ORF-9 in Scaffold_90, and ORF-7 and ORF-11 in Scaffold_56) were clustered with homologs from various fibrolytic bacteria. The other 2 GH3 enzymes (encoded, by ORFs 28–29 and ORF-30, respectively, in Scaffold_90) were identified as Bgl3D and Bgl3E (both are beta-glucosidases), because they clustered with Bgl3D and Bgl3E of *Butyrivibrio proteoclasticus* B316 and *Ruminococcaceae* bacterium D16. In addition, both had homologs in *Marvinbryantia formatexigens* DSM14469 and *Ruminococcus gnavus* ATCC29149. It was noteworthy that Bgl3D and Bgl3E in the reference genomes were encoded by 2 adjacent genes, bgl3D and bgl3E, as were our 2 GH3 enzymes encoded by adjoining ORFs.

Other identified carbohydrate-associated genes included those coding for 3 sugar transporters (ORF-9 and ORF-10 in Scaffold_56, and ORF-10 in Scaffold_90), a sugar isomerase (ORF-13 in Scaffold_56) and a sugar kinase (ORF-14 in Scaffold_56) (Figure 3 and Additional file 5). All 3 sugar transporters were suger-cation symporters which catalyze the uptake of simple sugars, including galactosides, pentosides, and hexuronides, in conjunction with a monovalent cation (H^+^ or Na^+^). According to the BLAST results, the isomerase and kinase were probably associated with utilization of L-arabinose and/or D-xylose, and participated in pentose and glucuronate interconversions. Furthermore, 5 genes that encoded transcriptional regulators (ORF-8 of Scaffold_56 and ORF-8, 11, 26, and 32 of Scaffold_90) may be involved in regulation of gene expression associated with carbohydrate utilization, due to their proximity to carbohydrate metabolism genes.

## Discussion

Based on the metagenomic profile of cecal microbiota, the giant flying squirrel underwent profound changes to adapt it to a diet of high-fiber, low-quality leaves. As reported for other small herbivores [1], the prominent cecum of the giant flying squirrel is apparently an anaerobic chamber for microbial breakdown of plant materials, consistent with an important role for cecal microbiota. It is noteworthy that cecal microbiota of the flying squirrel differed from their functional counterpart (rumen microbiota) of cattle (which has been much better characterized). Furthermore, the microbiota of the flying squirrel were also different from those of the Prevost's squirrel and laboratory mice, although they are close relatives. In the case of the flying squirrel and cattle, we concluded that independent evolutionary routes lead to similar functions. However, in the case of the flying squirrel and two omnivorous rodents (the Prevost's squirrel and lab mice), the influence of diet apparently confounded the phylogeny. In particular, the present study, based on wild-caught mammals, represented gut microbial communities under natural conditions and contributed important new knowledge regarding intricate mechanisms and interactions of the mammalian "super-organism". Moreover, most studies on gut microbes have been based on fecal samples (“output” of the digestive system) that may not reflect the actual reactions and processes involved in digestion of foods (“input”). If the digestive tract is regarded as a “production line”, the present study of cecal microbiota could elucidate the true “power house” for liberation of energy from a diet that is generally resistant to digestion, and thus offer insights into processes shaped by evolution for use of novel energy sources.

Based on a comparison of gut microbiota of flying squirrels (hindgut fermenter) and cattle (foregut fermenter), these 2 animals have distinct bacterial compositions, although both rely on the microbiota for the conversion of plant materials into nutrients. They had different phylotypes within *Firmicutes*, *Actinobacteria*, *Proteobacteria*, and *Verrucomicrobia*. These differences might be driven by diet (tree leaves versus forage and legumes), gut physiology (cecum versus rumen), and co-evolution within 2 host lineages (*Rodentia* versus *Artiodactyla*). Also, the gut microbiota of the mouse and flying squirrel were compared, since both species are phylogenetic kins (Order *Rodentia*). On the basis of observations in mouse models [14,18], the relative abundance of *Firmicutes* and *Bacteroidetes* was associated with the capacity to harvest energy. Compared with lean mice, obese mice had a relatively high fermentative capability, which was associated with an increased number of *Firmicutes*[14,18]. Since the cecal microbiota of the flying squirrel contained a high percentage of *Firmicutes* and harbored many genes involved in carbohydrate metabolism, we inferred that this system might be efficient at extracting energy from dietary polysaccharides, as reported in obese mice [14,18].

In addition to the host digestive system, microbial genomes encoding proteins with metabolic functions are responsible for conversion of dietary substances into absorbable nutrients [19,20]. The present sequence-based study provided a comprehensive method to reconstruct the primary metabolic profile of the cecal microbiota which enables the flying squirrel to survive on a leaf-based diet. According to the metagenomic data, the 3 main aspects of this complex degradation system are: 1) Plant polysaccharides are broken down into monosaccharides and disaccharides by various microbial glycoside hydrolases, and these simple sugars are transported into bacterial cells and fermented into short-chain fatty acids (principally butyrate, acetate, propionate, and lactate), which provide energy for the gut epithelium and other tissues [1,21]. 2) Genes involved in protein biosynthesis were much more abundant than those in protein degradation, consistent with other herbivorous microbiomes [22]. Due to the low protein content of a leaf-based diet, the cecal microbiota of the flying squirrel require specialized mechanism to derive nitrogen from limited sources. In that regard, the cecal microbiome contained genes related to hydrolysis of urea (derived from the host) into ammonia for synthesis of amino acids and derivatives. 3) The cecal microbiota synthesizes several vitamins, especially B-complex vitamins, which may meet the host’s need for these compounds [23].

Although several studies have focused on polysaccharide utilization by gut microbiota [15,24-27], there is a paucity of knowledge regarding gut microbial constituents and their functional interactions with the host, especially in wild animals. According to the CAZy database, multiple enzymes with the ability to catabolize dietary carbohydrates were detected in the cecal microbiome of the flying squirrel. Presumably metagenomic studies on the microbiota of wild herbivores that consume a wide range of plants will provide further insights regarding conversion of plant polysaccharides into monosaccharides. Based on the distribution of CAZy families detected in our fosmid library, we inferred that enzymes for plant oligosaccharide degradation (GH2, GH3, GH29, GH35, and GH39) may be more vital than those for degradation of crystalline cellulose (GH9) in the cecum, because the digesta has already been substantially degraded by physical and chemical digestion before it reaches the cecum. Furthermore, based on functional annotations, it appeared that *Firmicutes* has an important role in hydrolyzing indigestible dietary polysaccharides, such as components of plant cell walls (e.g. cellulose, xylan and pectin) and undigested starch, consistent with previous reports [28,29].

In general, metagenomic samples from environments with a stable input and turnover of complex plant biomass have a higher abundance of GHs than those from other samples [30]. The GH homologs in our dataset accounted for approximately 1.5% of the total predicted genes, a similar to that reported in gut metagenomes from the termite, human, and mouse [30]. In addition, the present fosmid library contained more than 16 GH families that were highly diverse; this diversity was comparable to that in other cellulolytic bacterial genomes and metagenome datasets [15,31,32]. In general, sequence-based searches are more efficient than function-based screening in prospecting for novel enzymes, since target genes can be directly discovered from metagenomic datasets using bioinformatics tools [33]. Although metagenomic approaches were used to quickly annotate various carbohydrate-active enzymes, functional assays will be required for confirmation, since sequence homology does not guarantee functional identity. Considerable additional studies are required to further elucidate and characterize the diverse plant biomass-degrading genes of the cecal microbiome.

High-throughput sequencing has been used to generate numerous gene candidates for biocatalysts; thereafter, their enzymatic activities have been characterized, with a substantial proportion of putative GHs having predicted enzyme activities [34]. However, most sequence-based metagenomic studies have limitations for downstream cloning and expression of genes, since the coverage is not enough to assemble full-length ORFs, due to the high microbial complexity of most environmental samples [30]. We therefore constructed a fosmid library, in which each clone contained an insert of ~40 kb of genomic sequence, long enough to reveal the cluster of genes in a genome, thereby improving characterization of the cecal microbiome. In this study, 2 fosmid inserts representing a total of 60 ORFs were identified as genomic fragments of *Firmicutes*, the most abundant and diverse phylum among the mammalian indigenous microbial communities [13]. These 2 inserts contained large gene clusters associated with plant polysaccharide utilization, including transcriptional regulators, glycoside hydrolases, sugar transporters, and downstream genes. The genomic arrangement of these 2 fragments verified that genes of associated metabolic pathways typically clustered together [35]. In prokaryotes, functionally related genes tend to form operons; conservation of neighboring genes suggested co-regulation and co-expression [36]. Based on sequence comparison, our results confirmed co-occurrence of Bgl3D and Bgl3E in several bacterial genomes, consistent with a functional interaction between this pair of GH3 enzymes.

## Conclusions

We characterized cecal microbiota of the flying squirrel, a small wild rodent with unique dietary preferences. On the basis of functional profiles, we inferred that microbial metabolism greatly contributed to intestinal functions, including degradation of carbohydrates, metabolism of proteins, and synthesis of vitamins. Furthermore, since 33 polysaccharide-degrading enzymes and 2 large genomic fragments containing a series of carbohydrate-associated genes were identified, we concluded that cecal microbiota have great metabolic potential for converting diverse plant materials into absorbable nutrients. Although the present study was based on metagenomic analysis of a limited number of samples, these findings are a valuable first-step exploration of cecal microbial diversity and functions in wild-caught flying squirrels. Further screening of novel enzymes degrading plant polysaccharides and metatranscriptomic analysis could enhance our knowledge of how plant biomass is processed by wild folivorous animals, in association with their symbiotic microbial community.

## Methods

### Sample collection and intestinal measurements

Five mature Formosan white-faced flying squirrels (*Petaurista alborufus lena*), 2 males (FS1 and FS5) and 3 females (FS2, FS3 and FS4), were collected from the mountains of Taiwan, where this species is common and not protected. The collecting permit (No. 0990007029) was granted by Yushan National Park Headquarters. Sampling (collection of specimens and tissues) and experiments were conducted in accordance with the Wildlife Conservation Act [37]. Body weight and length of FS1-FS4 were determined. The weight and length (with contents included) of the small intestine, cecum, and large intestine, were dissected from their mesentery, laid in a straight line, and measured with a 30-cm ruler. Immediately thereafter, cecal contents were removed and placed in RNAlater (Applied Biosystems, Foster City, CA, USA) for further processing. Flying squirrels FS1 and FS2 were used for 16S rRNA gene library construction, whereas FS3 and FS4 were used for analyzing food bolus particle size (data not included). The cecal sample of FS5 was preserved in RNAlater immediately after death to provide abundant, high-quality DNA for characterizing the cecal metagenome.

### DNA extraction of gut microbes

Cecal contents were centrifuged (14,000 x g for 10 min) to remove RNAlater and re-suspended in PBS solution. The suspension was prefiltered through 20-μm nylon net filters (Millipore, Bedford, MA, USA) to trap large debris, followed by a series of filters (12-, 10-, 8-, and 5- μm Isopore membrane filters; Millipore) to remove eukaryotic cells, and the filtrate was centrifuged (14,000 x g for 10 min) to pellet prokaryotic cells. High molecular weight DNA was extracted using Wilson's protocol [38], but with an additional 30 min of lysozyme digestion (5 mg/mL final concentration) at 37°C to lyse prokaryotic cells.

### Construction, sequencing, and phylogenetic analysis of 16S rRNA gene libraries

Two 16S rRNA gene libraries of cecal samples from 1 male (FS1) and 1 female (FS2) were constructed. The PCR reaction was performed using universal bacterial primers 8F (AGAGTTTGATCMTGGCTCAG) and 1492R (GGYTACCTTGTTACGACTT) with *Ex Taq* polymerase (Takara, Shiga, Japan), under the following conditions: 94°C for 2 min; 25 cycles of 94°C for 30 s, 54°C for 30 s, 72°C for 2 min, and finally 72°C for 10 min. The PCR products were ligated into the yT&A vector (Yeastern, Taipei, Taiwan) and transformation of *E. coli* was performed according to the manufacturer’s instructions. Positive colonies (n = 1,000) were picked and sequenced using ABI BigDye Terminator on ABI 3730xl *sequencers* (Applied Biosystems) and sequences were trimmed and edited using the Sequencher program (Gene Code Corporation, Ann Arbor, MI, USA). A total of 960 partial sequences (> 700 bp) were aligned and clustered into Operational Taxonomic Units (OTUs) based on their sequence similarity. Chao1 diversity and rarefaction were generated using the QIIME pipeline [39]. For comparison, 16S rRNA gene sequences from fecal samples of mammals [13], the mouse cecum [14] and the bovine rumen [15] were used for analyses of bacterial composition and community clustering. Statistical differences in proportions of bacterial phylum were determined by Pearson's chi-square test (*Χ*^2^). A distance-matrix for large alignments was created using the PHYLIP-DNADIST program [40], based on Jukes-Cantor models of nucleotide evolution. A phylogenetic tree was generated using FastTree [41] for UniFrac analyses [42] embedded in the QIIME pipeline [39].

### Fosmid library construction and sequencing

A fosmid library of the cecal sample from FS5 was constructed using a CopyControl™ Fosmid Library Production Kit and the pCC2FOS™ vector (Epicentre, Madison, WI, USA), according to the manufacturer’s instructions. Insert sizes of randomly selected fosmid clones were determined by *Not*1 restriction and gel electrophoresis. A total of 4,704 fosmid clones (average insert size, 30–40 kb) were obtained, representing a total of approximately 188 Mb of metagenomic fragments. End-sequences from all fosmid clones were obtained by Sanger sequencing from 1 end, using the T7 primer (TAATACGACTCACTATAGGG) on ABI 3730xl *sequencers* (Applied Biosystems). Inserts of 100 randomly selected fosmid clones were subjected to paired-end pyrosequencing (Genome Sequencer FLX System, Roche/454 Life Sciences, Branford, CT, USA).

### Analyses of fosmid end-sequences

Fosmid end-sequences were trimmed with SeqTrim software [43]; a total of 3,473 high-quality sequences were obtained for further analyses. Open reading frames (ORFs) were assigned using MetaGeneMark [44] and annotated by MG-RAST ( [45] using a cut-off value < 1e-5, according to the SEED subsystems [16]. Detailed information regarding protein functions and classifications was provided by similarity searches using BLAST (cut-off value < 1e-5) in the following databases: (1) NCBI nr database [46]; (2) eggNOG database [47]; (3) KEGG database [48]; and (4) Pfam database [49]. In addition, carbohydrate-active enzymes were detected with a CAZymes Analysis Toolkit [50] using sequence-based (cut-off value < 1e-40) and Pfam-based (cut-off value < 1e-5) annotation, according to the CAZy database [17].

### Analyses of fosmid inserts containing carbohydrate-associated genes

Pyrosequencing paired-end reads were assembled into community metagenomes using the GS *De Novo* Assembler program (Roche/454 Life Sciences). Assembly of 157 Mb raw sequences resulted in 125 scaffolds, with the average coverage = 52 and the N50 scaffold length = 38 kb. The ORF prediction and annotation for each scaffold were performed as described in the analysis of fosmid end-sequences. A search for non-coding RNA sequences was performed against an in-house database compiled from the following databases: SILVA [51], RDP [52], Greengenes [53], and Rfam [54]. In addition, a search for tRNAs was performed using tRNAscan-SE [55]. In this dataset, two large scaffolds (both > 30 kb), each containing 3 carbohydrate-active enzymes, were chosen for further analyses. A distance dendrogram of protein-coding genes was reconstructed with the neighbor-joining method (1,000 bootstrap replications) using MEGA 5 software [56].

### GenBank accession numbers

The following gene sequence data were uploaded to GenBank: 16S rRNA [JQ335999-JQ336958]; fosmid end-sequence [JS583577-JS587049]; and fosmid insert sequence [JQ335997-JQ335998].

## Abbreviations

CAZy: Carbohydrate-Active enZYmes; CBM: Carbohydrate-binding modules; CE: Carbohydrate esterase; COG: Clusters of Orthologous Groups of proteins; GH: Glycoside hydrolase; GI: Gastrointestinal; KEGG: Kyoto Encyclopedia of Genes and Genomes; MG-RAST: Meta Genome Rapid Annotation using Subsystem Technology; NCBI-nr: Non-redundant protein database of National Center for Biotechnology Information; ORF: Open reading frame; OTU: Operational Taxonomic Unit; PCoA: Principal coordinates analysis; Pfam: Database of protein families.

## Competing interests

The authors declare that they have no competing interests.

## Authors' contributions

HP Lu, CH Hsieh and HT Yu conceived the study design; HP Lu and HT Yu collected the samples; HP Lu, SW Huang, and M Wu did the molecular experiments and sequencing; YB Wang, CY Lin and M Wu carried out the bioinformatics analyses; and HP Lu and HT Yu wrote the first draft. All authors contributed to data interpretation and preparation of the final manuscript. All authors read and approved the final manuscript.

## Supplementary Material

Additional file 1 Rarefaction curves of observed species (a) and Chao1 diversity (b). Click here for file

Additional file 2 **Principal Coordinates Analysis (PCoA) based on the UniFrac metric comparing the phylogenetic composition of various gut microbiota.** FS1 and FS2 represent cecal microbiota of 2 flying squirrels; M1, M2, and M3 represent cecal microbiota of 3 mice [14]; C8, C64, and C71 represent rumen microbiota of 3 cattle [15]. Click here for file

Additional file 3 Functional categories of the cecal microbiota of the flying squirrel, according to the COG database. Click here for file

Additional file 4 Functional categories of the cecal microbiota of the flying squirrel, according to the KEGG database. Click here for file

Additional file 5 Putative functions and taxonomic assignments of predicted ORFs of 2 fosmid inserts: Scaffold_56 (GenBank: JQ335997) and Scaffold_90 (GenBank: JQ335998). Click here for file
