# Supplementary figures and images for: Metagenomic analysis reveals a functional signature for biomass degradation by cecal microbiota in the leaf-eating flying squirrel (Petaurista alborufus lena)
Source: BMC Genomics. 2012 Sep 10;13:466. doi: 10.1186/1471-2164-13-466 (PMC3527328; doi:10.1186/1471-2164-13-466)

## Additional File 1.

Rarefaction curves of observed species (a) and Chao1 diversity (b)

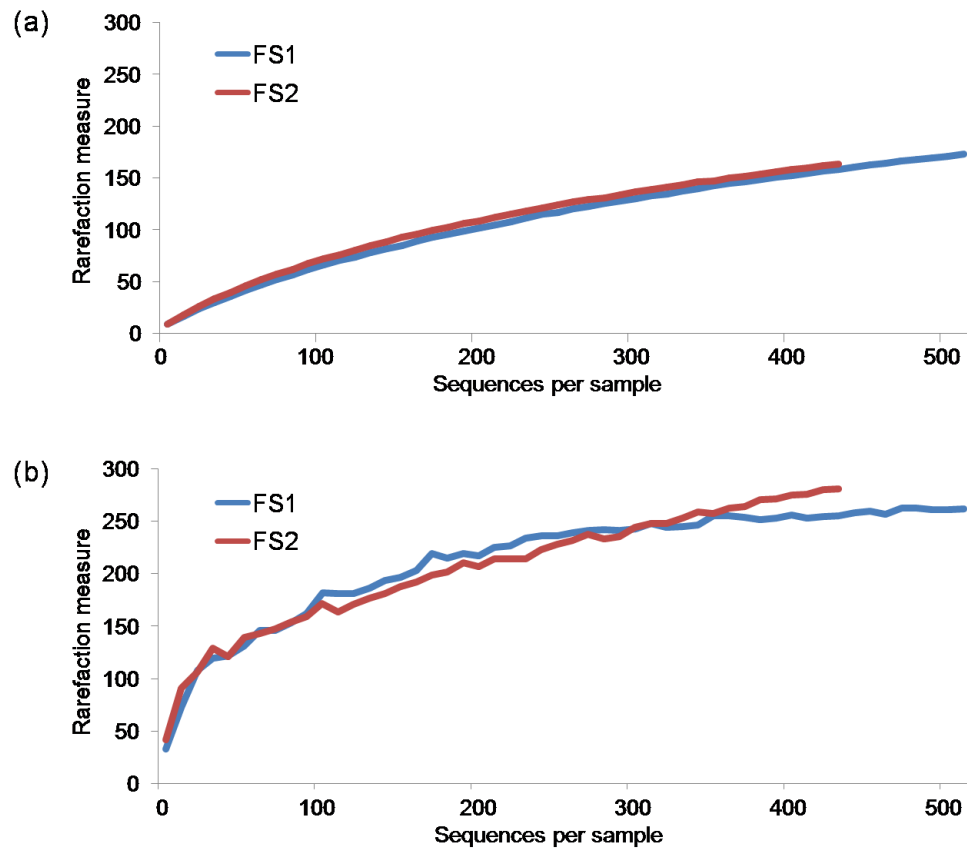

Supplement: Additional file 1 — Rarefaction curves of observed species (a) and Chao1 diversity (b). [file 1471-2164-13-466-S1.pdf]
