# Supplementary material for: Metagenomic analysis reveals a functional signature for biomass degradation by cecal microbiota in the leaf-eating flying squirrel (Petaurista alborufus lena)
Source: BMC Genomics. 2012 Sep 10;13:466. doi: 10.1186/1471-2164-13-466 (PMC3527328; doi:10.1186/1471-2164-13-466)

Additional file 2.

Principal Coordinates Analysis (PCoA) based on the UniFrac metric comparing the phylogenetic composition of various gut microbiota. FS1 and FS2 represent cecal microbiota of 2 flying squirrels; M1, M2, and M3 represent cecal microbiota of 3 mice [14]; C8, C64, and C71 represent rumen microbiota of 3 cattle [15].

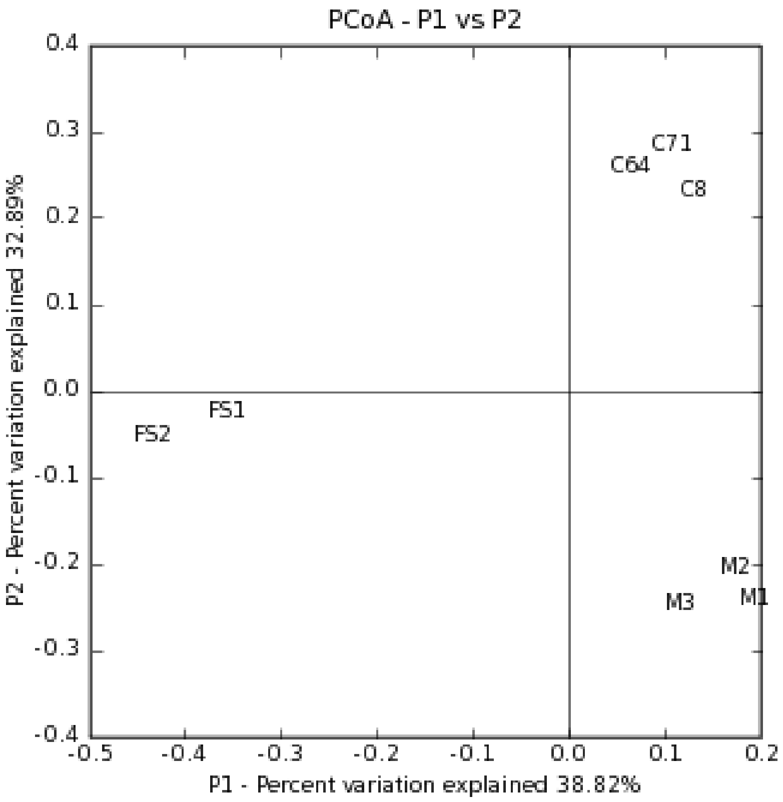

Supplement: Additional file 2 — Principal Coordinates Analysis (PCoA) based on the UniFrac metric comparing the phylogenetic composition of various gut microbiota. FS1 and FS2 represent cecal microbiota of 2 flying squirrels; M1, M2, and M3 represent cecal microbiota of 3 mice [14]; C8, C64, and C71 represent rumen microbiota of 3 cattle [15]. [file 1471-2164-13-466-S2.pdf]
