# Supplementary material for: Metagenomic analysis reveals a functional signature for biomass degradation by cecal microbiota in the leaf-eating flying squirrel (Petaurista alborufus lena)
Source: BMC Genomics. 2012 Sep 10;13:466. doi: 10.1186/1471-2164-13-466 (PMC3527328; doi:10.1186/1471-2164-13-466)
Supplement: Additional file 3 — Functional categories of the cecal microbiota of the flying squirrel, according to the COG database. [file 1471-2164-13-466-S3.pdf]

Additional file 3.

Functional categories of the cecal microbiota of the flying squirrel, according to the COG database.

Code and description of functional categories	No. hits	(%)
Information storage and processing	760	(17.86)
L: Replication, recombination and repair	268	(6.29)
J: Translation, ribosomal structure and biogenesis	253	(5.94)
K: Transcription	230	(5.41)
A: RNA processing and modification	6	(0.13)
B: Chromatin structure and dynamics	4	(0.09)
Cellular processes and signaling	959	(22.54)
M: Cell wall	289	(6.79)
T: Signal transduction mechanisms	168	(3.95)
O: Posttranslational modification, protein turnover, chaperones	154	(3.63)
V: Defense mechanisms	142	(3.34)
U: Intracellular trafficking, secretion, and vesicular transport	115	(2.71)
D: Cell cycle control, cell division, chromosome partitioning	55	(1.29)
N: Cell motility	20	(0.47)
W: Extracellular structures	9	(0.20)
Z: Cytoskeleton	7	(0.16)
Y: Nuclear structure	0	(0.00)
Metabolism	1,375	(32.32)
E: Amino acid transport and metabolism	299	(7.02)
C: Energy production and conversion	287	(6.75)
P: Inorganic ion transport and metabolism	219	(5.14)
G: Carbohydrate transport and metabolism	177	(4.15)
H: Coenzyme transport and metabolism	154	(3.61)
F: Nucleotide transport and metabolism	123	(2.88)
I: Lipid transport and metabolism	81	(1.91)
Q: Secondary metabolites biosynthesis, transport and catabolism	37	(0.87)
Poorly characterized	1,161	(27.29)
S: Function unknown	656	(15.42)
R: General function prediction only	505	(11.86)
