# Supplementary material for: Metagenomic analysis reveals a functional signature for biomass degradation by cecal microbiota in the leaf-eating flying squirrel (Petaurista alborufus lena)
Source: BMC Genomics. 2012 Sep 10;13:466. doi: 10.1186/1471-2164-13-466 (PMC3527328; doi:10.1186/1471-2164-13-466)
Supplement: Additional file 4 — Functional categories of the cecal microbiota of the flying squirrel, according to the KEGG database. [file 1471-2164-13-466-S4.pdf]

**Additional File 4.**

Functional categories of the cecal microbiota of the flying squirrel, according to the KEGG database.

| **Code and description of functional categories** | **No. hits** | **(%)** |
|---|---|---|
| Metabolism | 3,124 | (73.7) |
| 1.1 Carbohydrate metabolism | 564 | (13.31) |
| 1.2 Energy metabolism | 245 | (5.78) |
| 1.3 Lipid metabolism | 114 | (2.69) |
| 1.4 Nucleotide metabolism | 255 | (6.02) |
| 1.5 Amino acid metabolism | 554 | (13.07) |
| 1.6 Metabolism of other amino acids | 93 | (2.19) |
| 1.7 Glycan biosynthesis and metabolism | 93 | (2.19) |
| 1.8 Metabolism of cofactors and vitamins | 179 | (4.22) |
| 1.9 Metabolism of terpenoids and polyketides | 70 | (1.65) |
| 1.10 Biosynthesis of other secondary metabolites | 63 | (1.49) |
| 1.11 Xenobiotics biodegradation and metabolism | 143 | (3.37) |
| 1.12 Overview | 751 | (17.72) |
| Genetic information processing | 502 | (11.84) |
| 2.1 Transcription | 21 | (0.5) |
| 2.2 Translation | 146 | (3.44) |
| 2.3 Folding, sorting and degradation | 103 | (2.43) |
| 2.4 Replication and repair | 232 | (5.47) |
| Environmental information processing | 346 | (8.16) |
| 3.1 Membrane transport | 231 | (5.45) |
| 3.2 Signal transduction | 108 | (2.55) |
| 3.3 Signaling molecules and interaction | 7 | (0.17) |
| Cellular processes | 122 | (2.88) |
| 4.1 Transport and catabolism | 18 | (0.42) |
| 4.2 Cell motility | 28 | (0.66) |
| 4.3 Cell growth and death | 70 | (1.65) |
| 4.4 Cell communication | 6 | (0.14) |
| Organismal systems | 69 | (1.63) |
| Human diseases | 76 | (1.79) |
