# Supplementary material for: Metagenomic analysis reveals a functional signature for biomass degradation by cecal microbiota in the leaf-eating flying squirrel (Petaurista alborufus lena)
Source: BMC Genomics. 2012 Sep 10;13:466. doi: 10.1186/1471-2164-13-466 (PMC3527328; doi:10.1186/1471-2164-13-466)
Supplement: Additional file 5 — Putative functions and taxonomic assignments of predicted ORFs of 2 fosmid inserts: Scaffold_56 (GenBank: JQ335997) and Scaffold_90 (GenBank: JQ335998). [file 1471-2164-13-466-S5.pdf]

Additional File 5.

Putative functions and taxonomic assignments of predicted ORFs of 2 fosmid inserts: Scaffold_56 (GenBank: JQ335997) and Scaffold_90 (GenBank: JQ335998).

ORF	Putative function (COG category if available)	Closest relative (Organism / Phylum)	Size (aa)	GC (%)
Scaffold_56				
1	Uncharacterized vancomycin resistance protein (V)	*Clostridium ramosum / Firmicutes*	417	63
2	Sigma 54 modulation protein / SSU ribosomal protein S30P (J)	*Ruminococcaceae bacterium / Firmicutes*	48	63
3	Hypothetical protein	No significant similarity found	76	63
4	ATP-dependent metalloprotease FtsH (O)	*Ruminococcaceae bacterium / Firmicutes*	732	65
5	Tyrosine--tRNA ligase (J)	*Subdoligranulum variabile / Firmicutes*	409	64
6	Integrase family protein (L)	*Ruminococcaceae bacterium / Firmicutes*	134	58
7	Beta-glucosidase-related glycosidases (G)	*Roseburia intestinalis / Firmicutes*	810	60
8	Transcriptional regulator, TetR family (K)	*Clostridium acetobutylicum / Firmicutes*	191	50
9	Na+/melibiose symporter and related transporters (G)	*Marvinbryantia formatexigens / Firmicutes*	195	54
10	Na+/melibiose symporter and related transporters (G)	*Marvinbryantia formatexigens / Firmicutes*	365	59
11	Beta-glucosidase-related glycosidases (G)	*Ruminococcus torques / Firmicutes*	890	60
12	Beta-galactosidase / beta-glucuronidase (G)	*Marvinbryantia formatexigens / Firmicutes*	771	60
13	L-arabinose isomerase (G)	*Subdoligranulum variabile / Firmicutes*	111	53
14	Sugar (pentulose and hexulose) kinases (G)	*Roseburia intestinalis / Firmicutes*	512	64
15	Toxin-antitoxin system, toxin component, PIN family (R)	*Eubacterium saphenum / Firmicutes*	137	47
16	Addiction module antitoxin, RelB/DinJ family (L)	*Butyrivibrio fibrisolvens / Firmicutes*	94	48
17	Transposase, IS4 family (L)	*Ruminococcus albus / Firmicutes*	472	53
18	Transposase, IS4 family (L)	*Ruminococcus albus / Firmicutes*	430	53
19	SH3 domain protein (T)	*Clostridium cellulolyticum / Firmicutes*	264	65
20	Hypothetical protein	No significant similarity found	77	65
21	Conserved hypothetical protein	*Ruminococcaceae bacterium / Firmicutes*	78	61
22	Hypothetical protein	No significant similarity found	32	60
23	IS186 transposase (L)	*Escherichia coli / Proteobacteria*	371	52
24	Hypothetical protein	No significant similarity found	62	62
25	Hypothetical protein	No significant similarity found	72	66
26	Conserved hypothetical protein (S)	*Eubacterium dolichum / Firmicutes*	992	59
27	Prophage protein (R)	*Coprobacillus sp. / Firmicutes*	364	65
28	Prophage protein (S)	*Coprobacillus sp. / Firmicutes*	213	63
Scaffold_90				
1	Branched-chain amino acid transporter AzlC (E)	*Eubacterium yurii / Firmicutes*	58	47
2	NAD-dependent protein deacetylases, SIR2 family (K)	*Ruminococcus albus / Firmicutes*	286	47
3	Conserved hypothetical protein (R)	*Jonquetella anthropi / Synergistetes*	98	43
4	Nitroreductase (C)	*Ruminococcus sp. / Firmicutes*	195	51
5	NADPH:quinone reductase / Zn-dependent oxidoreductases (C)	*Synergistetes bacterium / Synergistetes*	334	51
6	Predicted Fe-S oxidoreductases (R)	*Bacteroides pectinophilus / Bacteroidetes*	328	50
7	Hypothetical protein	No significant similarity found	48	59
8	Transcriptional regulator, HxlR family (K)	*Butyrivibrio proteoclasticus / Firmicutes*	111	44
9	Beta-glucosidase-related glycosidases (G)	*Cellulosilyticum ruminicola / Firmicutes*	835	51
10	Na+/melibiose symporter and related transporters (G)	*Marvinbryantia formatexigens / Firmicutes*	469	47
11	Transcriptional regulator, AraC family (K)	*Marvinbryantia formatexigens / Firmicutes*	412	44
12	Conserved hypothetical protein (S)	*Bacteroides capillosus / Bacteroidetes*	207	48
13	Putative chlorophyll synthesis pathway, BchC (E)	*Clostridium hathewayi / Firmicutes*	338	52
14	Transcriptional regulator, TetR family (K)	*Clostridium hathewayi / Firmicutes*	197	48
15	Hypothetical protein	No significant similarity found	75	54
16	Hypothetical protein	No significant similarity found	103	44
17	Conserved hypothetical protein (S)	*Butyrivibrio proteoclasticus / Firmicutes*	488	46
18	Conserved hypothetical protein (S)	*Roseburia inulinivorans / Firmicutes*	176	47
19	Conserved hypothetical protein (S)	*Holdemania filiformis / Firmicutes*	399	48
20	Conserved hypothetical protein (S)	*Clostridiales bacterium / Firmicutes*	366	42
21	NADH-dependent butanol dehydrogenase A, Fe-dependent (C)	*Clostridium methylpentosum / Firmicutes*	396	56
22	Integrase family protein (L)	*Clostridium scindens / Firmicutes*	411	48
23	Integrase family protein (L)	*Desulfotomaculum acetoxidans / Firmicutes*	324	50
24	Integrase family protein (L)	*Desulfotomaculum acetoxidans / Firmicutes*	348	47
25	4-carboxymuconolactone decarboxylase (S)	*Marvinbryantia formatexigens / Firmicutes*	264	50
26	Transcriptional regulator, MerR family (K)	*Clostridium methylpentosum / Firmicutes*	133	51
27	Conserved hypothetical protein	*Bifidobacterium breve / Actinobacteria*	203	48
28	Beta-glucosidase-related glycosidases (G)	*Ruminococcus gnavus / Firmicutes*	619	56
29	Beta-glucosidase-related glycosidases (G)	*Marvinbryantia formatexigens / Firmicutes*	221	55
30	Beta-glucosidase-related glycosidases (G)	*Marvinbryantia formatexigens / Firmicutes*	1076	54
31	Conserved hypothetical protein	*Marvinbryantia formatexigens / Firmicutes*	436	45
32	Response regulator of the LytR/AlgR family (K)	*Marvinbryantia formatexigens / Firmicutes*	97	46
